# On the use of composite analyses to form physical hypotheses: An example from heat wave – SST associations

**DOI:** 10.1038/srep29599

**Published:** 2016-07-14

**Authors:** Ghyslaine Boschat, Ian Simmonds, Ariaan Purich, Tim Cowan, Alexandre Bernardes Pezza

**Affiliations:** 1 School of Earth Sciences, The University of Melbourne, Victoria, Australia; 2 Australian Research Council’s Centre of Excellence for Climate System Science, Australia; 3 CSIRO Oceans and Atmosphere, Aspendale, Victoria, Australia; 4 School of Geosciences, University of Edinburgh, Edinburgh, United Kingdom; 5 Greater Wellington Regional Council and Victoria University of Wellington, Wellington, New Zealand

## Abstract

This paper highlights some caveats in using composite analyses to form physical hypotheses on the associations between environmental variables. This is illustrated using a specific example, namely the apparent links between heat waves (HWs) and sea surface temperatures (SSTs). In this case study, a composite analysis is performed to show the large-scale and regional SST conditions observed during summer HWs in Perth, southwest Australia. Composite results initially point to the importance of the subtropical South Indian Ocean, where physically coherent SST dipole anomalies appear to form a *necessary* condition for HWs to develop across southwest Australia. However, sensitivity tests based on pattern correlation analyses indicate that the vast majority of days when the identified SST pattern appears are overwhelmingly *not* associated with observed HWs, which suggests that this is definitely *not a sufficient* condition for HW development. Very similar findings are obtained from the analyses of 15 coupled climate model simulations. The results presented here have pertinent implications and applications for other climate case studies, and highlight the importance of applying comprehensive statistical approaches before making physical inferences on apparent climate associations.

Statistical analyses are commonly used in climate science as they provide deep insights into the variability of climate occurrences at various spatial and time scales. Composite analyses, in particular, are recognized as being a simple and effective tool to identify conditions observed during specific states of the climate. They can point to connections between a phenomenon and key surrounding regions^1^,^2^ and provide valuable information for hypotheses to be formed as to the physical mechanisms that may be involved in these connections. However, testing the actual significance of these hypothesized connections or unravelling the causality in these relationships^3^ often requires a combination of various statistical methods, and above all needs to be supported by an appropriate physical understanding of the climate system itself.

This study highlights the caveats of using composite analyses to form physical hypotheses on the associations between environmental variables. These caveats are illustrated using the example of the apparent links between heat waves (HWs) and regional sea surface temperatures (SSTs). This is an important and timely example given that in recent decades HWs have become more prevalent in many regions, *e.g.* across Europe^4^,^5^,^6^, North America^7^, China^8^ and Australia^9^,^10^. These events have increased in frequency, duration and intensity during the 20^th^ century^11^,^12^,^13^ and climate models project these trends to continue under the influence of climate change^14^,^15^,^16^,^17^. Across Australia, HWs are responsible for more deaths than any other natural hazard^18^, the inherent susceptibility to drought conditions making the country particularly vulnerable to the impacts of extreme heat. It is therefore of great interest to the community and other affected sectors to understand the factors contributing to HWs, and as such is an area of much research attention.

SSTs have been identified as key players in HW development in many regions across the globe. For example, SSTs in the Mediterranean and Black Seas have been shown to be closely correlated with eastern European HWs, as they can maintain or reinforce upper level anticyclonic flow^19^,^20^. Tropical SSTs and convection in the Atlantic Ocean have also been associated with Rossby wave train formation and atmospheric blocking conditions that can lead to summer HW formation across Europe and Russia^5^,^21^,^22^. Warm SST anomalies in the north Atlantic and northeast Pacific during boreal spring are also thought to have contributed to record heat extremes during the severe Dust Bowl drought over central United States in the 1930s^23^. A recent study has also found that HWs in eastern United States are associated with characteristic SST patterns in the extratropical Pacific and that these anomalies may provide skill in predicting HWs at lead times up to 50 days^24^. For Australia there are suggestions that SSTs in the Indian Ocean or over the Tasman and Coral Seas may contribute to the onset of HWs across the southwest and southeast regions by interacting with local atmospheric circulations^25^,^26^,^27^. However, the limited evidence provided so far by observations and the lack of agreement in models simulating these observed SST patterns^28^ suggest that further work is needed to understand the role of SSTs for Australian HWs, especially as the global ocean continues to warm^29^.

In light of these considerations, we explore the association between summer HWs in Perth, southwest Australia, and large-scale and regional SSTs, as an *illustrative* case study of the use of composite analyses. The data and methods are described in the first section and results from composite and correlation analyses are then presented. The final section discusses some caveats when interpreting the composite results, and more generally highlights the importance of combining different statistical approaches before forming physical hypotheses regarding climate connections.

## Methods

### Classifying heat waves

We take as our example of composite analysis the case of HW occurrences in southwest Australia and their relationship with SSTs in observations and in coupled climate models. For illustrative purposes, we have chosen the populated city of Perth^28^ for our case study and consider HW events during austral summer (December to February, DJF), the season when they have the greatest impact on human health, and can interact with other natural disasters such as bushfires^30^,^31^.

HWs can be defined as a period of consecutive days when conditions are excessively hotter than normal, with little relief at night^32^. The specific definition we adopt here is a period of three or more consecutive days, during which daily maximum temperature (T_max_) exceeds its monthly 90^th^ percentile, and daily minimum temperature (T_min_) also exceeds its monthly 90^th^ percentile on the second and third days. All days in a HW are referenced to the monthly threshold on the first HW day^25^. HW dates in Perth are determined using the daily T_max_ and T_min_ time series provided by the Australian Bureau of Meteorology station at Perth Airport (31.9°S, 116.0°E, WMO Number 94610) during 1948–2014. The use of daily station data helps preserve the small-scale spatial and temporal variability that is important for representing extremes, and the temperature threshold we chose is dependent on both the time and location of the station. HW dates were compared across (four) neighbouring stations in the southwest Australian region and despite the potential for the urban heat island effect to influence HWs in Perth, the list of HW events and composite patterns are robust across the stations (not shown).

To explore the potential association between these HW events and SST anomalies, we perform composite and correlation analyses using daily SST fields from the ERA-Interim reanalysis^33^, available from 1979 onwards at 1.5° spatial resolution. We calculate composites of daily anomalies (as a deviation from the 1979–2014 daily climatology for that specific calendar day), and the statistical significance of these composite anomalies is determined by a Monte-Carlo procedure^34^. This method describes the areas in the composite that depart significantly from the background variability in the available data, and for our purpose is considered more robust that the commonly-applied (parametric) Student’s *t* test^35^.

### SST composites and model experiments

Based on the significant SST anomalies from the composite maps, five key spatial regions are identified: the Southern Hemisphere (extending into the northern tropics, SH; [70°S-20°N]), the South Indian Ocean (SIO; [30°E-120°E; 55°S-15°S]), the West ([35°E-80°E; 38°S-52°S]), East ([80°E-120°E; 15°S-40°S]) and both West and East poles in the South Indian Ocean. To assess the relative importance of these large-scale and regional SSTs, pattern correlation coefficients are then calculated between daily summer SST anomalies and the observed SST composite over each domain. Results are fundamentally unchanged when we use SST anomalies in the week or month leading up to the HW, or other SST observational datasets (not shown).

Sensitivity tests are designed to test the physical implications from our composite analyses, using outputs from 15 coupled climate model simulations (Supplementary Fig. S1) from phase 5 of the Coupled Model Intercomparison Project (CMIP5)^16^,^28^. Daily T_max_ and T_min_ data from each model are interpolated to the Perth station location, and used to determine the dates of simulated HW events over the historical 1950–2005 period. SST composites for the first day of HWs are constructed for individual models, for the multi-model mean (shown in Supplementary Fig. S1), and compared to observations. Pattern correlations are then computed between daily SST anomalies from each model and the observed (ERA-Interim) SST composite over the previously selected domains to quantify the likelihood of a HW to be simulated during specific SST conditions.

## Results

### Selection of HW events

**Figure 1:**
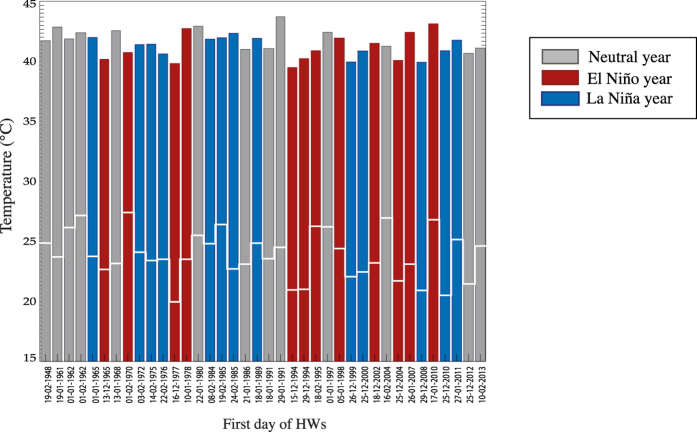
Bar plot of observed temperature extremes for each Perth HW event. Average T_max_ conditions over the first three HW days (*bar*) and average T_min_ conditions during the second and third of those days (*white line*). The colour of each bar shows the concurrent phase of El Niño Southern Oscillation during austral summer, based on a threshold of  ± 0.5 °C for the Oceanic Niño Index [3-month running mean of SST anomalies in the Niño 3.4 region (5^o^N-5^o^S, 120^o^-170^o^W)].

Using the above HW definition for observations, we detect 38 HW events for Perth during 1948–2014. Figure 1 lists the first day of each of these events, and shows the average T_max_ and T_min_ conditions recorded for the first three days of each event. The colour of each bar also indicates the concurrent phase of El Niño Southern Oscillation (ENSO) in the tropical Pacific during DJF, based on a threshold of ± 0.5 °C for the Oceanic Niño Index [three-month running mean of SST anomalies over the Niño 3.4 region (5^o^N-5^o^S, 120^o^-170^o^W)]. Overall, this figure illustrates the diversity that is observed in Perth HW characteristics, as T_min_ and T_max_ values vary quite substantially (especially T_min_ with ~2 °C standard deviation, possibly linked to the occurrence and timing of sea breezes in the area). The amplitudes of the bars suggest no obvious trend in the severity of HWs since 1948. However there is some indication of an increase in frequency of events, and indeed the mean of the observed HW years (*i.e.* 1987.5) differs significantly from the mean of all years of the study period (*i.e.* 1980) at the 95% confidence level [using a *t* test to reject the null hypothesis of no statistical significance difference between the means^36^]. Figure 1 also suggests no obvious relationship between the occurrence of HWs in Perth and ENSO during 1948–2014 (with 12 occurring during El Niño, 13 in La Niña and 13 in neutral years), consistent with recent findings from Perkins *et al.*^37^ (their Fig. 2a).

### Formation of composites

**Figure 2:**
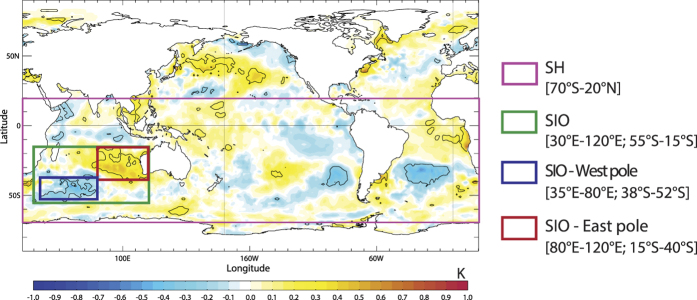
Composite SST anomalies for the first day of Perth HWs observed over 1979–2014. (The 25 HW events are listed across the *x*-axis in Fig. 1.) Composite anomalies that are significant at the 90% confidence level using a Monte-Carlo procedure^34^ are shown in black contours. Boxes indicate key areas where SST indices (mean SST anomalies) are computed: over the Southern Hemisphere (SH, pink box), South Indian Ocean (SIO, green box) and SIO West (blue box) and East pole (red box) domains. This figure was produced with IDL version 8.1 software (2011, Exelis Visual Information Solutions, Boulder, Colorado, https://www.exelisvis.com/docs/whatsnew_in_8_1.html).

To explore the climate signature associated with observed HWs in Perth, SST composites are computed for the first day of each HW event during the 1979–2014 (satellite) period for which ERA-Interim data are available (*i.e.* the 25 most recent events listed in Fig. 1). The average anomalous SST conditions for these events are shown in Fig. 2, where statistically significant anomalies (at the 90% confidence level) are identified within black contours.

This composite suggests that HWs in Perth are associated with little or no significant SST signal over the tropics, consistent with the absence of a relationship with ENSO revealed in Fig. 1. A number of studies have shown that HWs occurring in midlatitude regions are more generally associated with extratropical wave-activity. In the case of southwest Australia, it has been suggested that HWs develop through Rossby wave amplification over the South Indian Ocean. A persistent high-pressure system then forms over the Great Australian Bight and is responsible for advecting warm continental air towards Perth and the surrounding coastline^25^,^28^. [Note that in summer there is a very high frequency of anticyclones in the Bight^38^.] In line with these previous studies, our composite results point to the importance of mid-to-high southern latitudes, and in particular of the subtropical South Indian Ocean where physically coherent SST dipole anomalies are observed (Fig. 2). This dipole has a southwest-northeast tilt and is characterized by cold SST anomalies in the southwest (southeast of southern Africa) and warm anomalies in the northeast (off the western coast of Australia).

The CMIP5 models are generally quite successful in capturing this SST dipole pattern in the South Indian Ocean, despite the different number of HWs considered for each model composite (Supplementary Fig. S1). The position and intensity of the dipole is remarkably well simulated by the multi-model mean (a pattern correlation of 0.78 with the observed composite over the SIO domain, see Supplementary Fig. S1), despite model-to-model variations in the spatial extent of the warm pole and amplitude of the cold pole. Interestingly, this SST pattern is also the most dominant signal in the multi-model mean composite, reflecting a consistent occurrence of this pattern across models despite the diverse and contrasting ENSO signals simulated in the tropical Pacific Ocean.

Overall, the location and structure of this SST dipole is consistent with the synoptic signature of a wave train propagating over the subtropical Indian Ocean. However, as such, the composite does not allow us to infer any causal relationship between HWs and SSTs. On one hand, the SST pattern could be viewed as a result of the anomalously low-pressure component of the wave train associated with Perth HWs over the subtropical Indian Ocean^25^. On the other, this dipole pattern could also be playing a key role in the onset of HWs, by contributing to the synoptic set up that is required for a HW to occur in Perth. Interestingly, this SST anomaly is similar to the negative phase of the Indian Ocean subtropical dipole (IOSD)^39^,^40^,^41^,^42^,^43^. [It should be noted that the structure of the SST composite between 80^o^-120^o^E, 0^o^-40^o^S in Fig. 2 is also the inverse of that which is strongly associated with ‘northwest cloudbands’ and cooler conditions across central Australia^44^. This is consistent with enhanced continental temperatures along the trajectory of air parcels that ultimately reach Perth as HWs]. The IOSD events have been shown to develop through local air-sea interactions from austral spring to summer, linked with the strengthening/weakening of the Mascarene High over the South Indian Ocean. During a negative dipole event (as observed in Fig. 2), a low-pressure anomaly is induced over the central South Indian Ocean due to a southward shift and strengthening of the subtropical high. The resulting anomalous southeasterly winds cause increased evaporation and upper ocean mixing, and thus SST cooling over the western pole, while reduced evaporation and latent heat loss associated with the northwesterly wind anomalies generate a warming over the eastern pole^45^,^46^. Our composite analysis (in Fig. 2) suggests that this subtropical SST dipole may form a *necessary* (seasonal) condition for HWs to occur in Perth.

In this specific case of HW-SST associations, we can ask whether this ‘statistically significant’ signal observed in the South Indian Ocean (and also present in the CMIP5 models) is sufficient to actually establish a link with HW occurrence. In other words, if HWs tend to occur during specific SST dipole conditions, does this necessarily (and reversibly) imply that SST plays a significant role in the occurrence and/or variability of HWs in Perth? The following analyses address these questions to determine the potential role and predictive power of SST for HWs in Perth.

### Correlation analyses

One way to explore this notion of reversibility and SST ‘predictive power’ is to evaluate whether the composite SST pattern shown in Fig. 2 is *specifically* linked to the occurrence of HWs in Perth or if it can also appear on ‘normal’ (non-HW) days. The approach we take is to assess how often summer SST conditions ‘resemble’ the composite SST pattern, and determine whether when this occurs it leads systematically to a HW in Perth.

To quantify the degree of resemblance, we calculate the pattern correlation between each daily SST field in DJF and the composite SST pattern shown in Fig. 2, separately for each of the key domains. This test is performed using daily summer SST anomalies from observations during 1979–2014 (a total of 3150 days) and using the longer time series provided by the 15 CMIP5 model simulations during 1950–2005 (a net total of 75698 days). Results for observations are shown in Table 1, in terms of the number of days when the pattern correlation falls within bins of 0.1 width, for all days in DJF (black values) and for HW days only (green values). (Results for the model simulations are shown in a similar form in Supplementary Table S1.) The number of days obtained when the pattern correlation approaches 1.0 reflect how often during summer the daily SST anomalies would resemble the typical HW conditions over each domain.

As could be expected, this correlation analysis shows that SST conditions over the large SH domain are very rarely in the same configuration as the typical HW state shown in Fig. 2. Indeed, the pattern correlation between daily SST and the composite SST for this domain ranges from −0.4 to 0.5 but remains close to 0 for most days in DJF including HW days (with 45–50% of summer days between −0.1 and 0.1, see Table 1). Although this distribution is shifted towards slightly higher correlation values for the HW days, the SST for most of the HW events is still poorly correlated with the composite signal in Fig. 2 (between 0–0.3 pattern correlation).

Compared to the large SH domain, SST in the regional SIO domains exhibit higher correlation scores, with generally more (HW) days when pattern correlations exceed 0.5 (see last columns in Table 1). However, if SST anomalies have a stronger tendency to resemble the ‘typical HW’ pattern in the SIO domain, does this necessarily lead to the occurrence of a HW?

**Figure 3:**
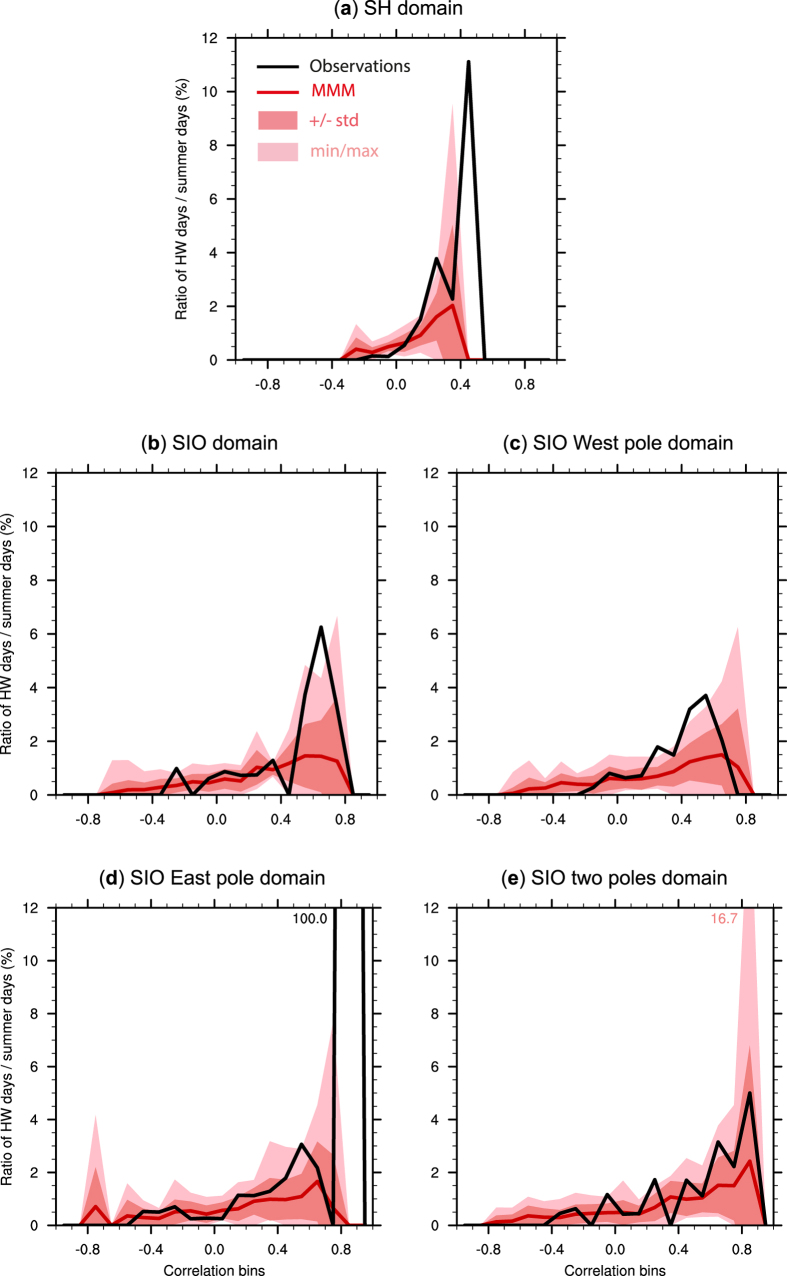
HW likelihood. Percentage ratio of HW days compared to total DJF days during which the pattern correlation between the Perth HW SST composite (Fig. 2) and daily SST anomalies falls within each 0.1 correlation bin, for the (**a**) SH, (**b**) SIO, (**c**) SIO-West pole, (**d**) SIO-East pole, and (**e**) SIO-two poles domains. The black curve shows the ratio calculated with daily SST anomalies from ERA-Interim (see values in Table 1). The pattern correlation is also calculated with daily SST anomalies from each CMIP5 model, and the red curve shows the multi-model mean (MMM) ratio. The dark (light) pink shading illustrates the ± 1 standard deviation (minimum and maximum) for all model ratios, with maximum values printed when exceeding 12%. Note that the values of some ratios for small domains can be misleading, *e.g.* the 100% ratio for observations over the SIO-East pole domain (**d**), is due to only one day with a high pattern correlation (>0.8) being a HW day (see Table 1).

Figure 3 illustrates the likelihood of a HW occurring in summer, given a specific resemblane or pattern correlation between daily SST and the composite HW state in Fig. 2. This likelihood is calculated as the ratio of HW days to all summer days for each correlation bin (the values presented in the two rows, for each domain, in Table 1), and plotted as a percentage for each domain and separately for observed and simulated SSTs. These plots indicate that even when daily SST anomalies over the SH domain strongly resemble the composite pattern in Fig. 2 (*e.g*. for correlations >0.5), HWs are very unlikely to occur (ratio close to 0 for both observations and model simulations, see Fig. 3a). In contrast, for the SIO domains, peak ratio values are obtained for higher correlation bins (between 0.6–0.7 correlation for the SIO domain and between 0.8–0.9 correlation for the domain centered on the two SIO poles, Fig. 3b,e), consistently for both observations and all model simulations. This suggests that HWs are *more likely* to occur when daily SST anomalies strongly resemble the SIO dipole configuration shown in Fig. 2, which is consistent with the initial inferences from our composite analysis. However, it should be noted that these peak values are quite small, and remain below 7% for observations and most models in the SIO domains (Fig. 3b,e) - with the exception of one model (MIROC-ESM-CHEM) simulating a maximum ratio of 16.7% in Fig. 3e. Therefore, although it may be more likely for a HW to occur when a SIO dipole-like SST pattern appears, this is still very rarely the case during summer. There are even a larger number of days in the season when this SST dipole appears (*i.e.* the correlation is high) but a HW does not occur (see Table 1).

## Discussion

This work highlights some caveats in using composite analyses to form physical hypotheses on climate connections, and we have presented a concrete example of this for the case of the relationship between HWs in Perth, southwest Australia and surface ocean temperatures. Results from our composite analysis initially suggest that SST dipole conditions in the South Indian Ocean may form a *necessary* condition for summer HWs to develop across southwest Australia. However, sensitivity tests based on pattern correlation analyses indicate that the vast majority of days when the SST pattern appears are *not* associated with HWs, which suggests that this is definitely *not a sufficient* condition. This example illustrates the non-reversibility of the HW-SST relationship observed in Fig. 2, as well as some of the limitations of forming physical hypotheses based solely on the use of composites.

Composites have been used in the literature as ‘indicators’ of the typical atmospheric and oceanic conditions accompanying specific climate states^25^,^27^. Composite patterns also carry useful climatic information, as they generally provide some interesting insight into forcing or connection pathways^47^. No assumption is made about the structure of the connection, since a composite analysis is by definition ‘non-parametric’^48^. Compared to other statistical methods (*e.g*. correlations), it has the advantage of taking into account the non-linearity of the climate system and therefore highlighting the asymmetry in certain relationships^49^. Many studies use these composite patterns as speculative basis to develop first hypotheses on potential climate connections and their underlying physical processes^50^. Carefully designed numerical experiments can then be conducted to test such hypotheses^51^.

However, while the composite approach appears straightforward, inconsistencies in the design, creation, interpretation and/or evaluation of composites can strongly affect the conclusions of some of these studies^52^,^53^. For instance, individual cases from the composite might occur under a different synoptic pattern, thereby raising the question whether these associations are statistically and physically robust. Various methods have been proposed to determine the statistical significance of composite patterns and account for this event-to-event variability. However, there is still much debate on the relevance and limitations of such statistical approaches, while fixating on statistical significance can also misdirect us from physically important processes^35^. More importantly, we need to bear in mind that the potential forcing or connection highlighted in composites has been ‘conditioned’ on a chosen subset of climate states, and this conditioned perspective is probably not reversible. The correlation analysis in our example is a clear illustration of this non-reversibility associated with composites. It shows that a statistically significant signal appearing during a specific state of the climate might appear as frequently, or even more frequently, during completely different states of the climate. In this case, correlations provide a simple and complementary angle to exploit the HW-SST association, along with valuable information to consider before making any physical interpretation of the initial composite patterns.

A number of studies have already pointed to the misinterpretation dangers inherent in many commonly used statistical techniques^3^,^53^,^54^. For example, Dommenget & Latif 55 have shown from a synthetic example that patterns derived from Empirical Orthogonal Function analyses can be misleading at times and associated with very little climate physics. In the case of the HW-SST association, it is salutary to keep in mind that the response of the atmosphere to oceanic forcing assumes rather different forms in the tropics and extratropics: the response to surface heating in the tropics occurs toward the ‘diabatic limit’, while in the higher latitudes advective terms become more important^56^. The atmospheric response to extratropical SSTs therefore tends to be much less vertically organised and more spatially dispersed, and very dependent on the larger scale circulation obtaining at the time. Our analyses seem to suggest that the atmospheric circulation patterns in the days and weeks preceding a HW can impact *on* the SST, and separately also culminate in a downstream midlatitude HW event. With this perspective there would be no direct connection between the mid-to-high latitude SST and HW occurrences, and model sensitivity experiments conducted with the identified Indian Ocean SST dipole would be expected to have limited success in simulating HWs. Indeed, we find the likelihood of a Perth HW when the dipole appears to be very small, in both the observations and model simulations.

In closing, we suggest that the message contained in the structure of composites may be quite misleading unless interpreted carefully, and that physical hypotheses framed from these should be rigorously tested with appropriate tools. By design, composites point to the possible importance of one parameter for a specific type of event, and SSTs have been the causal focus of this study. However extreme events are rarely attributed to a single physical “root cause”, and HWs have been shown to be influenced by other factors, including feedbacks between the atmosphere and surface related to antecedent soil moisture^57^,^58^, which are not discussed here. This HW-SST example serves as a simple case study and illustration of how much relevant climatic information can be extracted from composites and the limitations associated with our physical interpretation of these results.

## Additional Information

**How to cite this article**: Boschat, G. *et al.* On the use of composite analyses to form physical hypotheses: An example from heat wave – SST associations. *Sci. Rep.* **6**, 29599; doi: 10.1038/srep29599 (2016).

## Supplementary Material

Supplementary Information

## Figures and Tables

**Table 1:**
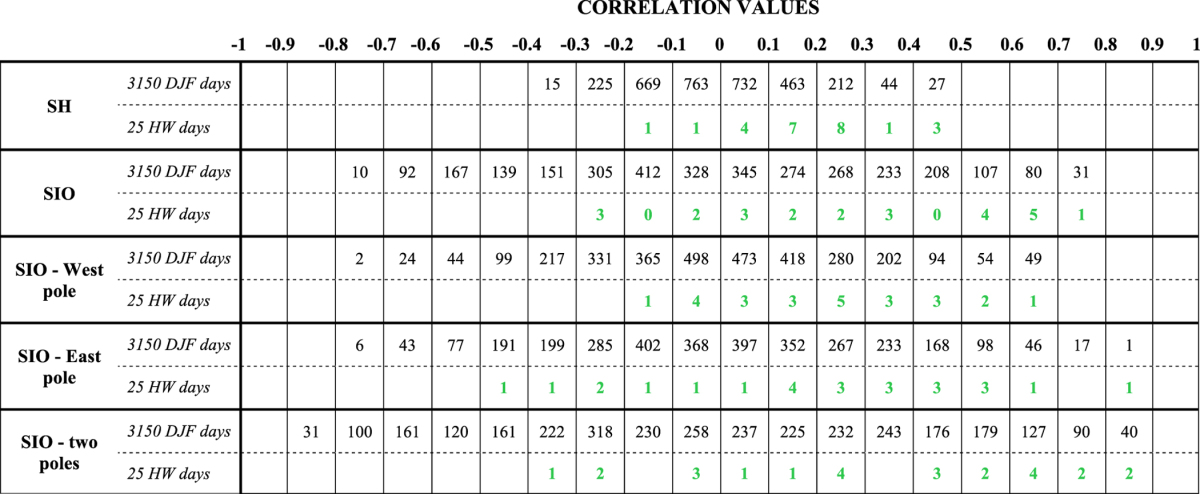
Frequency of the number of days when the pattern correlation between the Perth HW SST composite (Fig. 2) and daily SST anomalies falls within each 0.1 correlation bin, using ERA-Interim data.

Results are computed for the entire 3150 days in DJF (black values) and for the 25 HW days selected in DJF (bold italic) during 19792014, and are shown separately for the SH, SIO, SIO - West pole, SIO - East pole, and the SIO - two poles domains (shown in Fig. 2).
