# Erratum: On the use of composite analyses to form physical hypotheses: An example from heat wave – SST associations

**DOI:** 10.1038/srep31676

**Published:** 2016-08-24

**Authors:** Ghyslaine Boschat, Ian Simmonds, Ariaan Purich, Tim Cowan, Alexandre Bernardes Pezza

Scientific Reports 6: Article number: 2959910.1038/srep29599; published online: 07 14 2016; updated: 08 24 2016

This Article contains an error in the legend of Table 1.

“Results are computed for the entire 3150 days in DJF (black values) and for the 25 HW days selected in DJF (bold italic) during 1979–2014,”

should read:

“Results are computed for the entire 3150 days in DJF (black values) and for the 25 HW days selected in DJF (green values) during 1979–2014,”

