# Supplementary material for: On the use of composite analyses to form physical hypotheses: An example from heat wave – SST associations
Source: Sci Rep. 2016 Jul 14;6:29599. doi: 10.1038/srep29599 (PMC4944154; doi:10.1038/srep29599)
Supplement: Supplementary Information [file srep29599-s1.pdf]

SUPPLEMENTARY INFORMATION

On the use of composite analyses to form physical hypotheses: An example from heat wave – SST associations

Ghyslaine BOSCHAT[*1], Ian SIMMONDS[1],
Ariaan PURICH[2], Tim COWAN[3] and Alexandre Bernardes PEZZA[4]

CORRELATION VALUES

Domain		-1 to -0.9	-0.9 to -0.8	-0.8 to -0.7	-0.7 to -0.6	-0.6 to -0.5	-0.5 to -0.4	-0.4 to -0.3	-0.3 to -0.2	-0.2 to -0.1	-0.1 to 0	0 to 0.1	0.1 to 0.2	0.2 to 0.3	0.3 to 0.4	0.4 to 0.5	0.5 to 0.6	0.6 to 0.7	0.7 to 0.8	0.8 to 0.9	0.9 to 1
**SH**	*75698 days*							332	3182	11984	22805	21871	11521	3434	579						
	470 HW days								**13**	**34**	**111**	**142**	**106**	**53**	**11**						
**SIO**	*75698 days*			217	1239	2913	4906	6265	7308	7759	7962	7675	7195	6915	5986	4738	2993	1340	284	3	
	470 HW days				**2**	**7**	**10**	**18**	**26**	**38**	**36**	**46**	**38**	**70**	**57**	**56**	**41**	**21**	**4**		
**SIO - West pole**	*75698 days*			124	922	2808	4663	6541	7306	7814	8025	7864	7413	6773	6020	4842	3070	1338	171	4	
	470 HW days				**1**	**6**	**12**	**30**	**29**	**29**	**50**	**44**	**44**	**47**	**52**	**60**	**42**	**21**	**3**		
**SIO - East pole**	*75698 days*		3	340	1305	2977	4567	5745	6863	7621	8246	8313	7772	6965	5787	4671	2967	1334	220	2	
	470 HW days			**4**		**13**	**12**	**15**	**35**	**42**	**34**	**50**	**51**	**64**	**54**	**44**	**32**	**18**	**2**		
**SIO - two poles**	*75698 days*		459	2305	3949	4615	5070	5415	5436	5584	5504	5493	5230	5155	5269	5017	4655	3920	2091	531	
	470 HW days			**4**	**7**	**16**	**15**	**17**	**22**	**25**	**27**	**27**	**23**	**36**	**57**	**50**	**50**	**54**	**30**	**10**	

Supplementary Table S1: Frequency table showing the aggregate number of days when the pattern correlation between the Perth HW SST observed composite (Fig. 2) and daily simulated SST anomalies falls within each 0.1 correlation bin, separately for the SH, SIO, SIO-West, East and two poles domains (shown in Fig. 2). Results are computed for the all DJF days (black values) and for selected HW days (green values) in each model during 1950-2005, and then numbers are cumulated across all models.

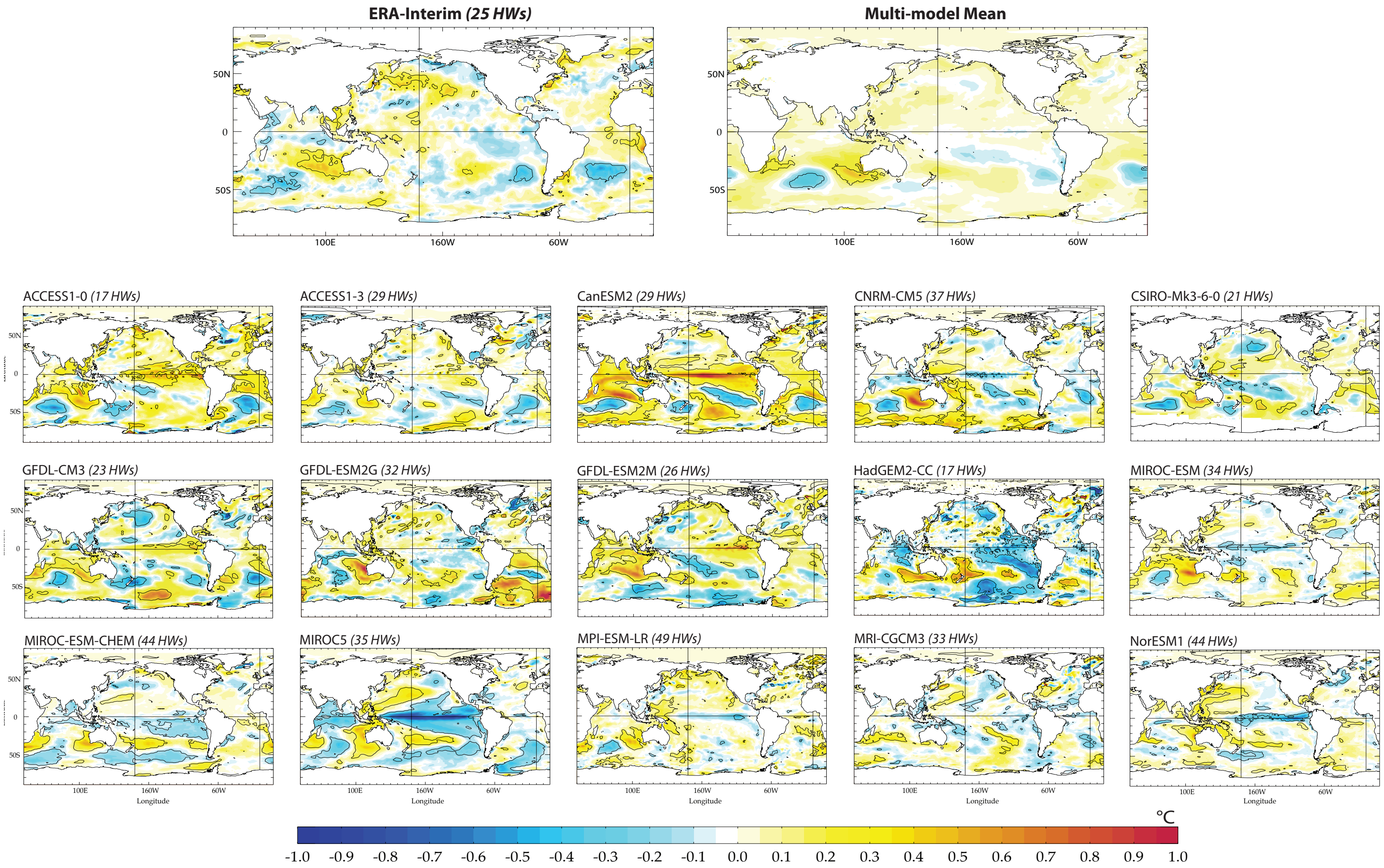

Supplementary Figure S1: Composite SST anomalies during the first day of Perth HWs simulated in each CMIP5 model over 1950-2005. The multi-model mean (MMM) is shown in the top panel, along with observations (ERA-Interim) over 1979-2014 for comparison. For each model, composite anomalies that are significant at the 90% confidence level using a Monte-Carlo procedure[34] are shown in black contours. In the MMM, each model is weighted evenly regardless of the number of HW events simulated, and regions are indicated where 80% of models agree on the sign of the MMM anomaly[28]. This figure was produced with IDL version 8.1 software (2011, Exelis Visual Information Solutions, Boulder, Colorado, https://www.exelisvis.com/docs/whatsnew_in_8_1.html).
